# Phlorotannins from Alaskan Seaweed Inhibit Carbolytic Enzyme Activity

**DOI:** 10.3390/md12105277

**Published:** 2014-10-22

**Authors:** Joshua Kellogg, Mary H. Grace, Mary Ann Lila

**Affiliations:** Plants for Human Health Institute, Department of Food, Bioprocessing and Nutrition Sciences, North Carolina State University, 600 Laureate Way, Kannapolis, NC 28081, USA; E-Mails: jjkellog@ncsu.edu (J.K.); mary_grace@ncsu.edu (M.H.G.)

**Keywords:** Alaska, seaweed, diabetes, glucosidase, hyperglycemia, amylase, type 2 diabetes mellitus, phlorotannin, polyphenol, ethnopharmacology

## Abstract

Global incidence of type 2 diabetes has escalated over the past few decades, necessitating a continued search for natural sources of enzyme inhibitors to offset postprandial hyperglycemia. The objective of this study was to evaluate coastal Alaskan seaweed inhibition of α-glucosidase and α-amylase, two carbolytic enzymes involved in serum glucose regulation. Of the six species initially screened, the brown seaweeds *Fucus distichus* and *Alaria marginata* possessed the strongest inhibitory effects. *F. distichus* fractions were potent mixed-mode inhibitors of α-glucosidase and α-amylase, with IC_50_ values of 0.89 and 13.9 μg/mL, respectively; significantly more efficacious than the pharmaceutical acarbose (IC_50_ of 112.0 and 137.8 μg/mL, respectively). The activity of *F. distichus* fractions was associated with phlorotannin oligomers. Normal-phase liquid chromatography-mass spectrometry (NPLC-MS) was employed to characterize individual oligomers. Accurate masses and fragmentation patterns confirmed the presence of fucophloroethol structures with degrees of polymerization from 3 to 18 monomer units. These findings suggest that coastal Alaskan seaweeds are sources of α-glucosidase and α-amylase inhibitory phlorotannins, and thus have potential to limit the release of sugar from carbohydrates and thus alleviate postprandial hyperglycemia.

## 1. Introduction

Type 2 diabetes is a chronic metabolic disease characterized by defects in insulin secretion and action, and has developed into a widespread global health threat. Currently, 20.9 million people in the United States are diagnosed with diabetes; nearly 8% of the adult population [1]. Postprandial hyperglycemia has been implicated as an important contributing factor in the development of insulin resistance [2], shown to be one of the first indicators of deteriorating glucose regulation [3]. In addition, postprandial glucose levels, compared against fasting glycemic response, have demonstrated stronger predictive power for diabetes-associated cardiovascular events [4], as well as other pathophysiological outcomes related to insulin resistance [5]. Hydrolysis of dietary starch is the foremost source of serum glucose, and the cleavage of glucose from carbohydrates is regulated by the activity of two main enzymes, α-amylase and α-glucosidase. Inhibition of these carbolytic enzymes reduces glucose absorption and the associated postprandial hyperglycemic spike.

Control of serum glucose by modulating the activity of α-amylase and α-glucosidase is one strategy in the management of diabetes [6,7]. Several approved antidiabetic pharmaceuticals—including acarbose, miglitol, and voglibose—exert their effects through this mechanism. However, these pharmaceuticals are non-specific inhibitors of both α-glucosidase and α-amylase, and inhibition of the latter releases larger starch fragments to the lower gastrointestinal tract that are not digestible by α-glucosidase [8]. Instead, they are digested by gut microbiota, resulting in adverse gastrointestinal effects similar to that of low-digestable carbohydrates (LDCs): diarrhea, abdominal cramping, and, in some cases, liver toxicity [9,10,11]. Thus, there is a continued need for natural α-amylase and α-glucosidase inhibitors that have greater specificity and fewer adverse secondary effects.

Seaweeds and their organic extracts contain a wide array of bioactive substances with diverse health benefits, including efficacy in counteracting metabolic syndrome and diabetes. *In vivo* assays using diabetic mice have demonstrated the efficacy of seaweed in improving fasting serum glucose levels [12]. Similar results have been shown in human clinical studies, where seaweed supplementation was correlated with increased insulin sensitivity [13], lowered glucose and triglyceride levels [14], and improved postprandial glycemic response [15]. One of the biochemical mechanisms responsible for the decrease in serum glucose levels is the inhibition of carbolytic enzymes. Seaweeds, especially their polyphenolic constituents, have exhibited strong inhibitory activity against both α-glucosidase and α-amylase [5,16,17,18,19].

The traditional diets and pharmacopeia of Native American/Alaska Native (NA/AN) populations have included coastal and benthic seaweeds for generations. Seaweeds have served as a source of macro- and micronutrients [20,21,22], and are featured in their ethnobotanical knowledge and dietary traditions. Nearly 60% of Inuit households in the Canadian Arctic’s Belcher Islands regularly consume *Rhodymenia* spp. and *Laminaria* spp. for example [23], and First Nations in British Columbia combine the red alga *Porphyra* with clams, salmon eggs, or fish into soups, as well as sprinkle dried seaweed over other foods [22]. However, over the last few decades, the dietary preferences of AN communities have shifted away from traditional subsistence diets to more commodity-based Western consumption, resulting in a diet that contains fewer traditional marine resources, including marine mammals and seaweeds [24,25,26,27]. This dietary evolution has been hypothesized as a contributing factor in the significant rise in diabetes incidence in these communities; AN populations are twice as likely to have diagnosed diabetes as non-Hispanic whites [28].

The cold, temperate oceans around Alaska hold an abundant diversity of macroalgae [29], yet little research has been undertaken to evaluate the ability of Alaskan seaweeds to influence hyperglycemia and carbolytic enzymatic efficacy. In this study, six species of seaweed harvested from the southern coast of Alaska were surveyed in order to identify seaweed extracts that hold potential for diabetic care through their inhibition of carbolytic enzyme activity.

## 2. Results and Discussion

### 2.1. Carbolytic Enzyme Inhibition

The inhibitory effect of Alaskan seaweed against α-glucosidase and α-amylase was determined using *p*-nitrophenyl-α-d-glucopyranoside (PNPG) and Lugol’s solution as colorimetric indicators, respectively. While previous studies to date have focused on a single species or class of macroalgae, this study evaluated the inhibitory effect of six seaweed species from the three distinct phyla of algae: Phaeophyta (*A*. *marginata*, *F*. *distichus*, *S*. *groenlandica*, and *S*. *latissima*), Rhodophyta (*P*. *fallax*), and Chlorophyta (*U*. *lactuca*). At 4 mg/mL, the crude extracts of the brown algae (*A. marginata* (AM), *F. distichus* (FD), *S. groenlandica* (SG) and *S. latissima* (SL)) significantly (*p* < 0.05) reduced both α-glucosidase and α-amylase activity (Table 1), and the red alga *P. fallax* (PF) only significantly impacted α-amylase activity. The two species, AM and FD, reduced enzyme activity to <20%, and were selected for subsequent fractionation.

**Table 1:** Inhibitory potential (% control) of Alaskan seaweed crude extracts ^#^.

Sample	Crude Extract Yield (g)	α-Glucosidase	α-Amylase
*Alaria marginata*	9.537	6.4 ± 0.8 ^a^ ***	17.9 ± 4.1 ^a^ ***
*Fucus distichus*	11.198	3.0 ± 1.2 ^a^ ***	18.4 ± 5.3 ^a^ ***
*Saccharina groenlandica*	11.595	76.1 ± 5.0 ^b^ ***	65.5 ± 3.2 ^b^ *
*Saccharina latissima*	13.011	75.1 ± 3.1 ^b^ ***	56.3 ± 9.8 ^b^ *
*Pyropia fallax*	7.780	86.6 ± 6.1 ^c^	62.2 ± 8.2 ^b^ *
*Ulva lactuca*	7.982	88.0 ± 6.7 ^c^	82.4 ± 9.4 ^c^

^#^ Samples were assayed at 4 mg/mL, data represents mean ± SEM (*n* ≥ 4); Different letters in same column denote significantly different values (*p* < 0.05); * *p* < 0.05 *vs.* uninhibited control; *** *p* < 0.001 *vs.* uninhibited control.

**Figure 1:**
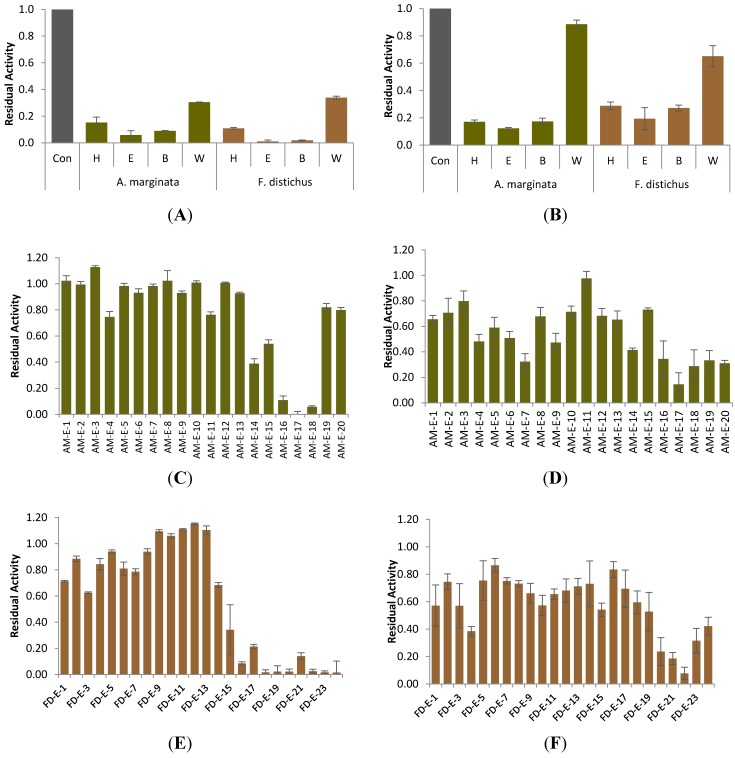
Inhibition of α-glucosidase (**A**) and α-amylase (**B**) by enriched partitions (4 mg/mL) of *F. distichus* and *A. marginata*; (**C**) and (**D**) inhibition of α-glucosidase and α-amylase, respectively, by subfractions (2 mg/mL) of AM-E; (**E**) and (**F**) inhibition of α-glucosidase and α-amylase, respectively, by subfractions (2 mg/mL) of FD-E. Con = control (untreated enzyme); H = hexane fraction; E = ethyl acetate fraction; B = 1-butanol fraction; W = water fraction. Bars indicate mean value ± SEM (*n* = 3).

Analysis of the organic partitions of AM and FD demonstrated that the medium-polar ethyl acetate fractions (AM-E and FD-E) were primarily responsible for the α-glucosidase and α-amylase inhibitory activity exhibited by the crude extracts (Figure 1A,B). An aliquot of 600 mg of AM-E was separated via flash silica gel chromatography, yielding 20 subfractions, while 1.1 g FD-E was charged to a Sephadex LH-20 column for separation, eluting 24 subfractions. Each subfraction was re-screened for inhibitory activity at an initial concentration of 2 mg/mL. From the AM-E subfractions, AM-E-17 (8.7 mg) displayed the greatest inhibition of α-glucosidase, reducing activity to 1.98% ± 0.14% of the control (Figure 1C), yet yielded moderate inhibition of α-amylase, with a residual activity of 14.44% ± 1.27% compared to the uninhibited control (Figure 1D).

From *F. distichus*, the polar subfractions demonstrated high activity levels against α-glucosidase and α-amylase. FD-E-22 (29.5 mg) evidenced highly effective inhibition of α-glucosidase, yielding 1.63% ± 0.55% of the control’s activity (Figure 1E). Similar to AM subfractions, FD-E-22 exhibited strong levels of α-amylase inhibition, reducing enzyme activity to 7.7% ± 0.12% of the control (Figure 1F).

### 2.2. Comparison of Inhibitory Activity with Acarbose

**Figure 2:**
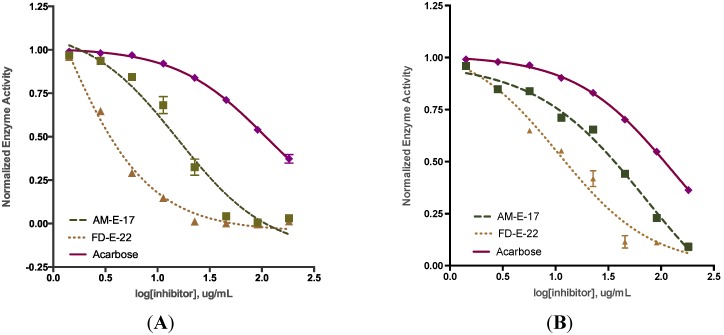
IC_50_ values for seaweed inhibition of α-glucosidase (**A**) and α-amylase (**B**). Extracts of *Alaria marginata* (AM (---)) and *Fucus distichus* (FD (…)) were evaluated at a range of concentrations (1.5–200 μg/mL final concentration); acarbose was used as a positive control.

The active subfractions of *A. marginata* and *F. distichus* displayed dose-dependent inhibition of α-glucosidase or α-amylase (Figure 2). The inhibitory activity of AM-E-17 and FD-E-22 was compared to that of acarbose, an oligosaccharide derived from *Actinoplane* spp. and widely known to inhibit both α-glucosidase and α-amylase. Table 2 shows the IC_50_ value for AM-E-17 and FD-E-22 for α-glucosidase and α-amylase inhibitory activity. The IC_50_ value for AM-E-17 and FD-E-22 inhibiting α-glucosidase was 15.66 ± 0.82 and 0.89 ± 0.08 μg/mL, respectively; significantly lower than that for acarbose. For this study, the IC_50_ of acarbose was determined to be 112.0 ± 2.85 μg/mL, similar in magnitude to other studies [19]. The greatly reduced values for the AM and FD fractions indicate they are efficient inhibitors of α-glucosidase.

Similarly, the two fractions of AM and FD that were most active at inhibiting α-glucosidase were highly active against α-amylase, and exhibited significantly lower IC_50_ values compared to acarbose. AM-E-17 inhibited α-amylase with an IC_50_ of 63.28 ± 0.87 μg/mL, while FD-E-22 had an IC_50_ value of 13.98 ± 1.32 μg/mL, compared to 138.7 ± 0.65 μg/mL for acarbose (Table 2). Both fractions evidenced stronger inhibitory activity than was observed with acarbose, implying increased efficiency of inhibition against α-amylase. However, α-glucosidase inhibitory activity (defined by each fraction’s IC_50_) was greater than for α-amylase, suggesting that the seaweed has preferential inhibition of α-glucosidase.

**Table 2:** IC_50_ values (μg/mL) for Alaskan seaweed inhibitory effect on α-glucosidase and α-amylase.

Sample ^a^	α-Glucosidase	α-Amylase
AM-E-17	15.66 ± 0.82 ***	63.28 ± 0.87 ***
FD-E-22	0.89 ± 0.08 ***	13.98 ± 1.32 ***
Acarbose	112.0 ± 2.85	138.7 ± 0.65

Data represents the mean ± SEM (*n* ≥ 4); ^a^ AM-E-17 = *Alaria marginata*’s ethyl acetate fraction, subfraction 17; FD-E-22 = *Fucus distichus*’ ethyl acetate fraction, subfraction 22; *** *p* < 0.001 *vs.* acarbose.

### 2.3. Inhibition Kinetics

**Figure 3:**
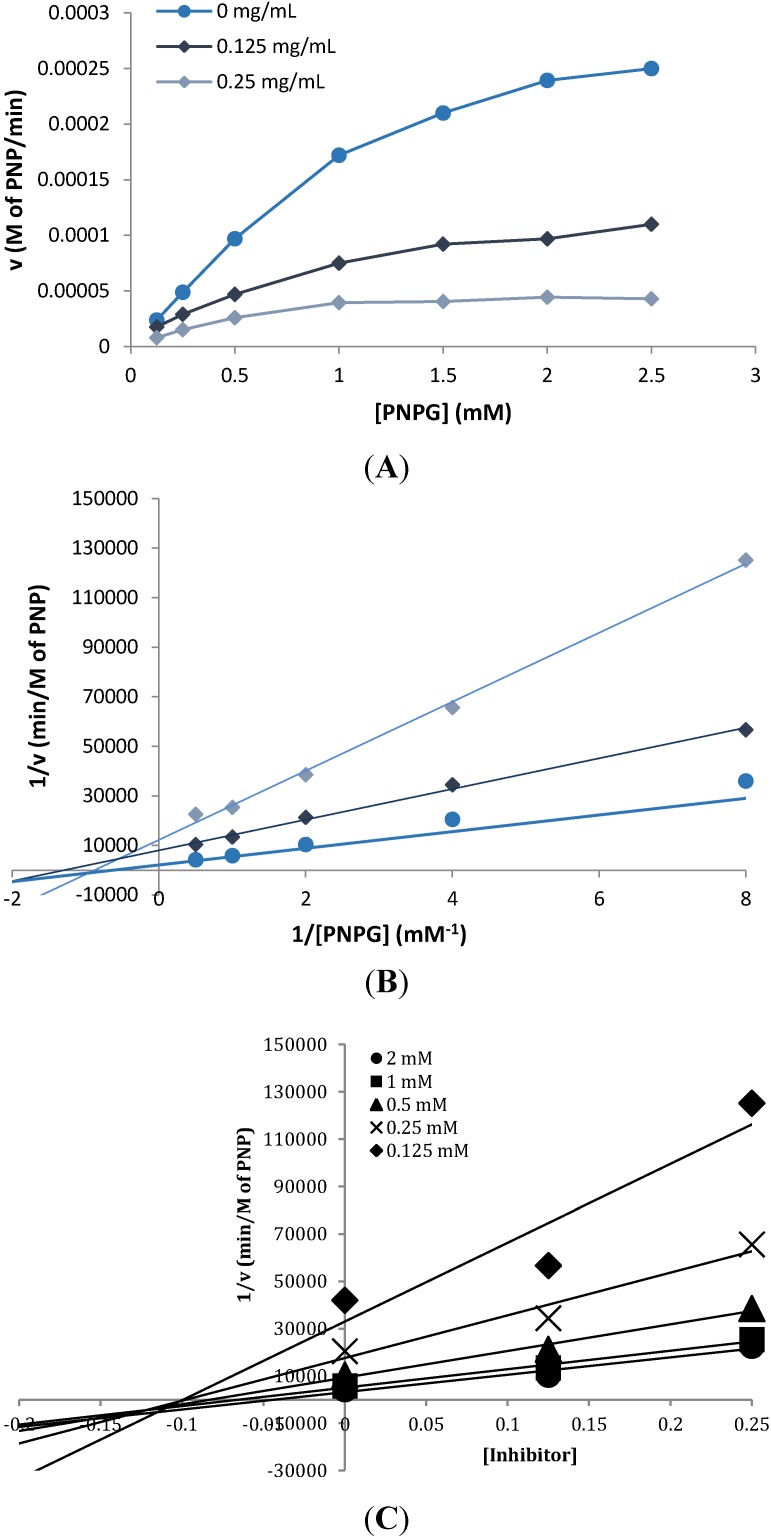
Kinetics of α-glucosidase inhibition in the presence or absence of *Fucus distichus*’ fraction FD-E-22 (●, 0 mg/mL; ♦, 0.125 mg/mL, ▲, 0.25 mg/mL). (**A**) Velocity *v* of carbolytic reaction at various concentrations of the substrate (*p*-nitrophenyl-α-d-glucopyranoside) (S); (**B**) Lineweaver-Burk plots for inhibition of α-glucosidase, the reciprocal of the velocity *versus* the reciprocal 1/(S); (**C**) Inhibition constant (K_i_ value) plot of 1/*v versus* (FD-E-22) at multiple [S] concentrations. Data points represent the mean value ± SEM (*n* = 3).

Kinetic studies of the inhibitory effect of FD-E-22 on α-glucosidase were performed using the same procedure as described above. The initial velocity *v* of the enzyme was measured at various concentrations of the substrate PNPG (Figure 3A). In the absence of any inhibitor, α-glucosidase had a Michaelis constant (K_m_) and V_max_ of 1.597 and 4.2 × 10^−4^ M/min, respectively, from the Lineweaver-Burk plot of the kinetic data. As shown in the plot (Figure 3B), the presence of FD-E-22 (0.25 mg/mL) lowered *v* and decreased K_m_ as well as V_max_ to 0.586 and 0.64 × 10^−4^ M/min, respectively. This suggested that FD-E-22 exhibited mixed mode inhibition against α-glucosidase, characterized by a combination of competitive and noncompetitive inhibition, which has been evidenced in other studies [17]. The inhibition coefficient K_i_ for FD-E-22 was shown to be 0.116 ± 0.009 mg/mL, determined by plotting 1/v against inhibitor concentration at each concentration of substrate (Figure 3C).

### 2.4. Phlorotannin Characterization

**Figure 4:**
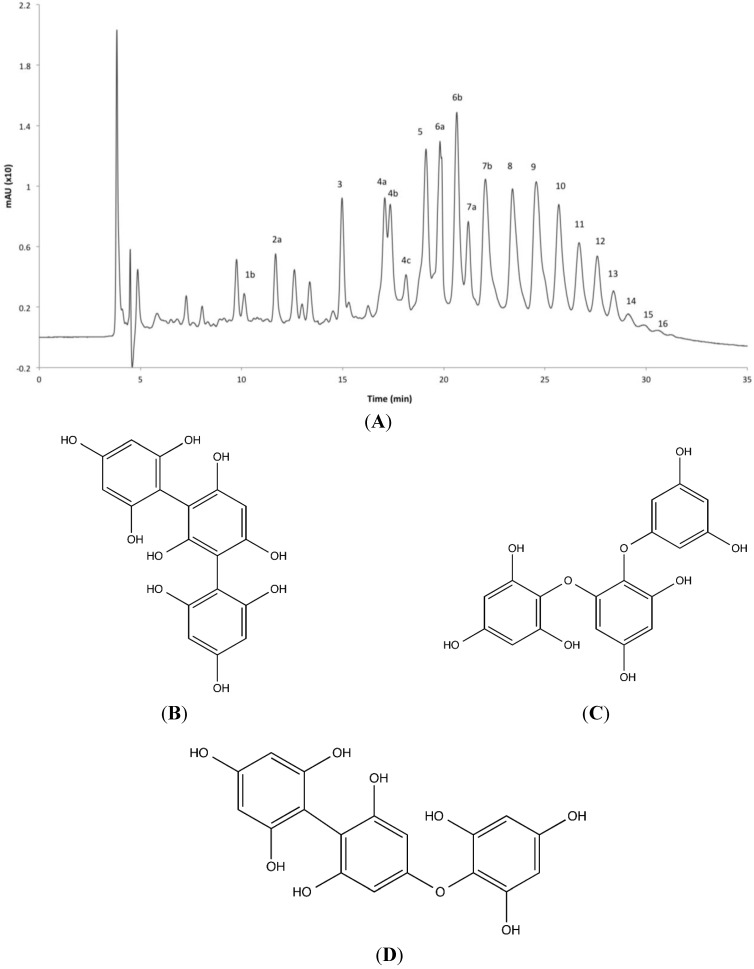
(**A**) LC-MS chromatogram for *Fucus distichus*’ FD-E-22 subfraction on a normal phase diol column with a binary mobile phase consisting of acetonitrile and 97:3 methanol:water. Peaks were measured at 254 nm. Peaks are labeled in order of elution (Table 3). (**B**–**D**) Representative structures of a trimeric fucol with aryl-aryl bonds (**B**); phloroethol showing ether bridges (**C**); and fucophloroethol with a mixture of the two linkages (**D**); all three possess identical accurate masses (374.0638 Da).

The ethyl acetate fraction of *F. distichus* exhibited the highest α-glucosidase and α-amylase inhibitory activity amongst the tested fractions, and had been shown in a previous study to possess the highest phenolic content of all fractions [30]. Sub-fractionating the active extract on Sephadex LH-20 yielded the potent extract FD-E-22, eluted with methanol: chloroform (4:1), which was subsequently explored to determine its chemical constituents. Reverse phase liquid chromatography was unsuited to separate the highly polar phlorotannin units. Instead, normal phase liquid chromatography (NPLC) with a diol column and a binary mobile phase consisting of acetonitrile and aqueous methanol allowed for separation of 24 phlorotannin isomers (Figure 4), comprising 95.4% of the fraction, determined from HPLC UV absorption at λ = 254 nm. These 24 isomers were analyzed via mass spectrometry, acquiring full spectra in both positive and negative ion modes from *m*/*z* 150 to 2500. Mass spectrometry analysis revealed an ion chromatogram with protonated molecular ions ([M + H]^+^) corresponding to fucophloroethol oligomers with three (375 Da) to 18 (2235 Da) phloroglucinol units. Masses corresponded to a single aryl or ether bridge between monomer units, as opposed to the heterocyclic dibenzodioxin and furan ringed phlorotannins, and, unlike fuhalols, there were no additional hydroxyl units on the phenyl rings. Phlorotannins were eluted sequentially by increasing degrees of polymerization (DP), forming successive peaks in the NPLC chromatogram with increasing ion mass.

The fragmentation of the parent peaks in both the positive and negative mass spectrometry mode provided a more comprehensive analysis of the phlorotannins present (Table 3). All analyzed compounds showed similar fragmentation patterns characteristic of fucophloroethol fragmentation, with losses of one and two molecules of water (−18 Da and −36 Da, respectively), phloroglucinol (−126 Da), the protonated molecular ion of phloroglucinol (−127 Da), as well as the tandem loss of phloroglucinol and methyl (−126 Da and –14 Da) (Table 3). Based on the MS/MS data, the 24 signals were categorized as fucophloroethol isomers ranging from three to 18 monomer units. Fucophloroethols are a group of phlorotannins exhibiting characteristics of both fucols and phloroethols, and possess combinations of aryl-aryl and ether-linked phloroglucinol units in either linear or branched arrangements; however, fucols, phloroethols, and fucophloroethols all have identical accurate masses and similar fragmentation patterns (Figure 4B,D), and mass spectrometry techniques are not sufficient to differentiate between them.

Several smaller phlorotannins (DPs of 4, 6, 7, 8, and 9) exhibited multiple ion peaks corresponding to the same *m*/*z* ion but differing in retention time on the column. This is possibly due to the presence of varying conformations of fucophloroethols having the same molecular ion yet differing in the branching position of the aryl and ether linkages of subsequent phloroglucinol additions [31,32].

The larger phlorotannins (DP > 11) manifested distinguishable peaks in the negative mode mass spectra as [M − H]^−^ and [M − 2H]^−2^. Mass spectrometry identification was achieved through the accurate mass parent ion and daughter ions (Table 3). The mass differences from fragmentation of these daughter ions were half of the corresponding mono-ionized fragmentation patterns; a single water loss was recorded as −9 Da, phlorotannin monomer unit loss −63 Da, and methyl group loss −7 Da. Nevertheless, each larger polymer evidenced cleavage into multiple daughter ions (Table 3), allowing for the confident characterization of these phlorotannins as fucophloroethols with DP from 11 to 18.

**Table 3:** Normal-phase liquid chromatography-mass spectrometry (NPLC)-IT-TOF-MS^2^ characterization of phlorotannins from FD-E-22 *.

Peak No.	RT (min)	Molecular Formula	ESI	Measured Mass (*m*/*z*)	Predicted Mass (*m*/*z*)	Δm (ppm)	DP ^a^	MS/MS Ions (*m*/*z*)					
1	9.752	C_18_H_14_O_9_	(+)	375.0710; [M + 1]^+^	375.0711	−0.1	3	357	231				
2a	11.860	C_24_H_18_O_12_	(+)	499.0907; [M + 1]^+^	499.0871	3.6	4	481	463	355	337	231	
2b	12.679	C_24_H_18_O_12_	(+)	499.0880; [M + 1]^+^	499.0871	0.9	4	481	463	355	338	231	
2c	12.996	C_24_H_18_O_12_	(+)	499.0880; [M + 1]^+^	499.0871	0.9	4	481	463	355	337	231	
2d	13.259	C_24_H_18_O_12_	(+)	499.0892; [M + 1]^+^	499.0871	2.1	4	481	463	356	337	231	
3	14.822	C_30_H_22_O_15_	(+)	623.1007; [M + 1]^+^	623.1031	−2.4	5	605	587	479	461	355	231
4a	16.255	C_36_H_26_O_18_	(+)	747.1121; [M + 1]^+^	747.1119	0.2	6	729	711	585	571	479	355
4b	16.943	C_36_H_26_O_18_	(+)	747.1135; [M + 1]^+^	747.1119	1.6	6	729	711	585	571	479	355
4c	17.345	C_36_H_26_O_18_	(+)	747.1089; [M + 1]^+^	747.1119	−3.0	6	729	711	585	571	479	355
5a	17.922	C_42_H_30_O_21_	(+)	871.1296; [M + 1]^+^	871.1328	−3.2	7	853	745	601	479		
5b	19.113	C_42_H_30_O_21_	(+)	871.1343; [M + 1]^+^	871.1328	1.5	7	853	745	601	479		
6a	19.809	C_48_H_34_O_24_	(+)	995.1530; [M + 1]^+^	995.1513	1.7	8	977	959	869	729	581	461
6b	20.632	C_48_H_34_O_24_	(+)	995.1532; [M + 1]^+^	995.1513	1.9	8	977	959	869	729	581	461
7a	21.204	C_54_H_38_O_27_	(+)	1119.1647; [M + 1]^+^	1119.1673	−2.6	9	1101	993	853	709	461	
7b	22.050	C_54_H_38_O_27_	(+)	1119.1716; [M + 1]^+^	1119.1673	4.3	9	1101	993	853	709	461	
8	23.390	C_60_H_42_O_30_	(+)	1243.1853; [M + 1]^+^	1243.1834	1.9	10	1225	959	851	469		
9	24.561	C_66_H_46_O_33_	(+)	1367.1960; [M + 1]^+^	1367.2000	−4.0	11	1351	1241	1227	705	683	
10	25.677	C_72_H_50_O_36_	(+)	1491.2123; [M + 1]^+^	1491.2155	−3.2	12	1473	1347	829	807	745	
11	26.681	C_78_H_54_O_39_	(−)	1613.2214; [M − 1]^−^	1613.2174	4.0	13	806	797	599			
12	27.581	C_84_H_58_O_42_	(−)	1737.2298; [M − 1]^−^	1737.2336	−3.8	14	868	859	806	797		
13	28.385	C_90_H_62_O_45_	(−)	1861.2540; [M − 1]^−^	1861.2496	4.4	15	931	922	868	859	735	643
14	29.113	C_96_H_66_O_48_	(−)	1985.2614; [M − 1]^−^	1985.2656	4.2	16	993	984	930			
15	29.905	C_102_H_70_O_51_	(−)	2109.2806; [M − 1]^−^	2109.2806	0.0	17	1055	1046	983			
16	30.578	C_108_H_74_O_54_	(−)	2233.2928; [M − 1]^−^	2233.2966	−3.8	18	1116	1107	1044	783	715	540

* FD-E-22 = *Fucus distichus*’ ethyl acetate fraction, subfraction 22; ^a^ Degree of polymerization.

Proton NMR yielded two groupings of signals. One represented the meta-distributed aromatic proton signals directly bound to the aryl rings (5.8–6.1 ppm). The second, broader due to proton exchanges, was seen in the hydroxyl proton range (9 ppm). The shift and multiplicity of these proton groupings was similar to other studies of phlorotannins [33], providing corroborating evidence to the NPLC-MS data suggesting the presence of fucophloroethol phlorotannins.

## 3. Experimental Section

### 3.1. Chemicals

Unless otherwise noted, all chemicals were of reagent or spectroscopic grade and obtained from Sigma-Aldrich (St. Louis, MO, USA).

### 3.2. Instrumentation 

LC–MS analysis was performed using a Shimadzu LC–MS-IT-TOF instrument (Shimadzu, Tokyo, Japan) equipped with a Prominence HPLC system (SIL-20A HT autosampler, LC-20AD pump system, SDP-M20A photo diode array detector). The LC separation was performed using a method adapted from Grace *et al.* [34] with a Develosil Diol column (250 mm × 4.6 mm × 5 μm, Phenomenex, Torrance, CA, USA) and a binary solvent system comprised of 0.2% acetic acid in acetonitrile (A) and methanol:water:acetic acid (97:3:0.2) (B). Compounds were separated into the ion source at a flow rate of 0.8 ml/min with the following step-wise gradient: 0%–40% B, 0–35 min; 40%–100% B, 35–40 min; 100% B, 40–45 min; 100%–0% B, 45–50 min; 0% B, 50–60 min. Prior to the next injection, the column was re-equilibrated for 5 min at initial conditions. The heat block and curved desolvation line (CDL) were maintained at 200 °C. Nitrogen was used as nebulizer and drying gas with the flow rate set at 1.5 L/min. The ESI source voltage was set at 4.5 kV and the detector was set at 1.5 V. The instrument was calibrated to <5 ppm error in mass accuracy with an external standard of sodium TFA solution. Ionization was performed using a conventional ESI source in positive and negative ionization mode. Data was acquired from *m*/*z* 150–2500. Shimadzu’s LCMS Solution software was used for system control and data analysis. NMR spectra were recorded on a Bruker Avance 700 MHz spectrometer (Bruker BioSpin Corporation, Billerica, MA, USA).

### 3.3. Sample Material

Seaweed samples of six Alaskan coastal species were harvested from the coastal area surrounding Sitka, Alaska in June 2012, including: four Phaeophyta (brown algae) species (*Fucus distichus*, * Saccharina latissima*, *Saccharina groenlandica*, and *Alaria marginata*); one species of Rhodophyta (red algae) (*Porphyra fallax*); and a species of Chlorophyta (green algae) (*Ulva lactuca*) (Table 4). Freshly collected specimens were rinsed to remove particulates and any epiphytes that might have attached to the surface, and transported to the laboratory. Samples were frozen at −80 °C, lyophilized, and kept at −80 °C until extract preparation.

**Table 4:** Alaskan seaweeds collected from Sitka, AK, USA June 19, 2012.

Phylum	Classification	Species	Abbreviation	Common Name
Phaeophyta	Brown seaweed	*Alaria marginata*	AM	Winged kelp
		*Fucus distichus*	FD	Bladder wrack
		*Saccharina groenlandica*	SG	Kelp
		*Saccharina latissima*	SL	Sugar kelp
Rhodophyta	Red seaweed	*Pyropia fallax*	PF	Laver
Chlorophyta	Green seaweed	*Ulva lactuca*	UL	Sea lettuce

### 3.4. Extraction and Isolation

40 g of each freeze-dried sample were powdered using a grinding mill (IKA, Wilmington, NC, USA) and the powder was suspended in 1 L 80% aqueous methanol and shaken overnight at room temperature, then filtered through Whatman #1 filter paper and the powder re-extracted a second time. The extract was evaporated under reduced pressure to remove excess solvent, and the resulting aqueous residue was diluted to 400 mL with deionized water and sequentially partitioned with hexane, ethyl acetate, and 1-butanol (3 × 400 mL), yielding 4 crude fractions including the aqueous residue (H, E, B, and W, respectively). Solvents were removed via rotary evaporation, and all fractions were lyophilized and held at −80 °C until analysis and subfractionation.

Active fractions were separated by flash chromatography using either silica gel (50 g, 230–400 mesh, 60 Å Merck, column dimensions 20 × 3 cm) eluted with hexane: ethyl acetate:methanol (100:0:0 → 0:50:50) to collect 20 subfractions, or Sephadex LH-20 columns (10 g dry weight, column dimensions 18 × 3 cm) eluted with methanol: chloroform 4:1 followed by 70% aqueous acetone to obtain 24 subfractions. The progress of separation was monitored by TLC (60 F_254_; Sigma-Aldrich, St. Louis, MO, USA) stained with vanillin-HCl.

### 3.5. Biochemical Assays

#### 3.5.1. α-Glucosidase Assay

A rapid, multi-well plate system was used to assay seaweed extracts and fractions for inhibitory activity against α-glucosidase. α-glucosidase from *Saccharomyces cerevisiae* (75 U/mg; Sigma-Aldrich, St. Louis, MO, USA) was diluted to a working concentration of 0.005 mg/mL in 100 mM phosphate buffer, pH 7 (PB). To each well of a 96-well plate, 20 µL of inhibitor, extract, or solvent (control) was mixed with 100 µL of the substrate *p*-nitrophenyl-α-d-glucopyranoside (PNPG; 1 mM in PB) and incubated at 30 °C for 5 min. 100 µL of the enzyme working solution was charged to each well, and absorbance was measured at 405 nm for 30 minutes using a Molecular Devices M3 microplate reader at 30 °C (Molecular Devices Inc., Sunnyvale, CA, USA). Blank wells (without enzyme) were subtracted from each well and results were compared *versus* a control (no inhibition). The commercial pharmaceutical acarbose was utilized as a positive control. Half-inhibitory concentration (IC_50_), where 50% of the enzyme activity was inhibited, was determined using serial dilutions of active extracts or fractions (*n* ≥ 6), and the percentage inhibition was plotted against the log_10_ sample concentration.

For each sample, the inhibitory activity was calculated as follows:

% inhibitory activity = (A_con_ − (A_ext_ − A_blank_))/A_con_ × 100% (1)

Where A_con_ is the absorbance of the uninhibited enzyme, A_ext_ is the absorbance of the enzyme treated with the extract, and A_blank_ is the absorbance of the extract with substrate (no enzyme present).

#### 3.5.2. Kinetics of α-glucosidase Inhibitors

The enzyme-inhibitor reaction was performed using the above method at both 0.25 and 0.125 μg/mL of the inhibitor and at substrate concentrations from 0.25 to 2 mM PNPG. The type of inhibition was determined by Lineweaver-Burk plot, using the double reciprocal of the substrate concentration and velocity of inhibition at *t* = 6 min. The inhibition constant Ki of the competitive inhibitor was calculated using the following equation:

1/v = K_m_ (1 + [inhibitor]/K_i_)/(V_max_[substrate]) + 1/V_max_ (2)

#### 3.5.3. α-Amylase Assay

To determine the inhibition of α-amylase, the α-amylase from porcine pancreas (Sigma-Aldrich, St. Louis, MO, USA) was dissolved in 20 mM Tris-HCl buffer pH 7.5 (Fisher Scientific, Pittsburgh, PA, USA) to a working concentration of 25 μM. Each well of a 96-well plate was charged with 35 μL of the extract, fraction, or inhibitor, with 5 μL of the starch substrate (1% w/v starch solution; Sigma-Aldrich), and incubated at 37 °C for 5 min. Ten microliters of the working enzyme solution was charged to each well, and the resulting solution was incubated for 10 min at 37 °C. 150 μL of diluted Lugol’s solution (1:1 dilution with distilled H_2_O) was added, and inhibition was determined by reading absorbance at 595 nm on a microplate reader. Blank wells without amylase were subtracted from each well to account for innate color of the sample. As with the α-glucosidase assay above, acarbose was utilized as the positive control, and IC_50_ values were generated from serially diluted sample solutions.

For each sample, the inhibitory activity was calculated as follows:

% inhibitory activity = (A_con_ − (A_ext_ − A_blank_))/A_con_ × 100% (3)

Where A_con_ is the absorbance of the uninhibited enzyme, A_ext_ is the absorbance of the enzyme treated with the extract, and A_blank_ is the absorbance of the extract with substrate (no enzyme present).

#### 3.5.4. Statistics

All assays were performed at least in triplicate. Results are presented as mean of triplicate runs ± SEM. Statistical analysis was conducted using repeated measures analysis of variance (ANOVA) followed by Tukey’s test (Prism 6.0, GraphPad Inc., La Jolla, CA, USA), with statistical significance determined at the *p* < 0.05 or *p* < 0.01 level. Half maximal inhibitory concentration (IC_50_) data was calculated after logarithmic transformation and expressed as the geometric mean with 95% confidence intervals. Lineweaver-Burk plots and kinetic data were obtained using biochemical algorithms in Prism.

## 4. Conclusions

The main approach of anti-diabetic treatments is the maintenance of circulating glucose concentrations to a near constant level by delaying absorption of glucose via inhibition of the two primary enzymes responsible for carbohydrate digestion, α-glucosidase and α-amylase. Due to undesirable side effects of synthetic inhibitors [9], there is a need for alternative anti-diabetic compounds, and certain polyphenols have proven to be particularly efficacious inhibitors of these two carbolytic enzymes [35]. Polyphenols are known to interact with a variety of proteins via hydrogen bonding and hydrophobic interactions, forming complexes which modulate enzymatic bioactivity [36]. Modeling the interaction between the active site of α-amylase and phenolic compounds, Piparo *et al.* [37] correlated the relationship between the inhibitory activity of flavonoids and the associated protein-polyphenol interactions. Their study postulated the dependence of inhibitory activity on the generation of hydrogen bonds between the hydroxyl groups of the polyphenols and the carboxylate groups of Asp197 and Glu233 located at the active site of α-amylase. While this study was based solely upon flavonoids from terrestrial plants, the presence of polyhydroxylated structures in phlorotannins could potentially allow the adoption of a similar conformation upon interaction with the enzyme active site, and thus inhibit α-amylase activity through a similar mechanism.

In this study, the organic fractions from six different seaweed species harvested from the southeastern coast of Alaska were evaluated for their potential to inhibit carbolytic enzyme activity. All species exhibited stronger inhibitory activity against α-glucosidase than α-amylase, which may suggest a more selective inhibition profile by seaweed extracts, with the potential to regulate the digestion and metabolism of complex carbohydrates without incurring the side-effects of non-specific enzyme inhibitors. Milder α-amylase inhibition has been shown to prevent abnormal gut microfloral fermentation of undigested carbohydrates when they enter the colon [9]. The four brown seaweed species exhibited inhibition of the enzymes, and two species, *A. marginata* and *F. distichus*, demonstrated sufficiently high activity against α-glucosidase and α-amylase to warrant further analysis using bioassay-guided fractionation techniques. From each of these two species, one fraction (AM-E-17 and FD-E-22) displayed efficacy as potential anti-hyperglycemic agents, and IC_50_ values for both demonstrated strong potential for the inhibition of α-glucosidase and α-amylase. The IC_50_ values for inhibition of α-glucosidase by FD-E-22 (0.89 ± 0.08 µg/mL) were significantly lower than reported values for isolated phlorotannin derivatives; one published study reported IC_50_ values for fucophloroethol and eckol oligomers ranging from 8.0 to 24.5 µg/mL [18], while a second study found eckol species inhibiting α-glucosidase with IC_50_ values from 1.2 to 17.8 µg/mL [38]. FD-E-22 possessed the greater inhibitory activity than AM-E-17, and was selected for more detailed phytochemical characterization.

The dominant phytochemicals in the active extract FD-E-22 were phlorotannins, polyphenolic oligomers of phloroglucinol. These polyphenolic polymers have been revealed in other members of the genus *Fucus*, especially in the widely studied *Fucus vesiculosus* and *F. spiralis* [31,32]. Phytochemical analysis of the fraction of *Alaria marginata* that demonstrated significant levels of enzyme inhibitory activity, AM-E-17, remained inconclusive. Originally hypothesized to also contain phlorotannins, none were observed after NPLC-MS analysis. This could be due to the initial use of silica gel for sub-fractionation of *A. marginata*, potentially leading to the oxidative breakdown of labile phlorotannins [31,39], whereas Sephadex LH-20 was employed for the fractionation of the FD-E partition. The inhibitory activity could have arisen from other phytochemicals present in the seaweed matrix, yet the mass spectrometry analysis was unable to provide a dominant molecular ion. Additionally, while the other genera of brown seaweed tested have been known to contain phlorotannins, including *Laminaria* and *Alaria*, it is possible that the concentration, degree of polymerization or configuration of phlorotannins were not sufficient to inhibit the assayed enzymes. Variations in structure of the polysaccharide fucoidan, isolated from both *Ascophyllum* and *Fucus*, displayed marked differences in the resulting enzyme inhibition of α-glucosidase and α-amylase, suggesting the potential for wide variation in these polymeric phytochemicals between genera [40].

An NPLC-MS methodology allowed for the separation and subsequent identification of phlorotannins up to 18 monomer units in size. Previous studies had reported the elution of phlorotannins greater than 1200 Da in size (a degree of polymerization 10 and higher), as a single, broad peak, with many isomers co-eluting simultaneously. This conglomeration of phlorotannins impeded accurate detection and characterization [39], and it was hypothesized that these large phlorotannins were either bound irreversibly to the column or unable to be individually eluted with the given conditions [31]. The column and solvent system employed in this study was sufficiently sensitive to discern multiple high molecular weight phlorotannins up to 18 phloroglucinol units, allowing for a more detailed analysis of larger polymer units.

In summary, traditionally consumed Alaskan seaweed, particularly brown kelps, evidenced a pronounced inhibitory effect on carbolytic enzyme activity. The phlorotannins found in *Fucus distichus* were even more effective in their inhibition efficacy than the known pharmaceutical inhibitor acarbose, suggesting their potential to delay the absorption of digested carbohydrates in the gastrointestinal tract *post*-consumption. Thus, these species have potential as natural sources of anti-diabetic agents that could reduce post-prandial hyperglycemia.

## References

[1] Centers for Disease Control & Prevention (CDC) Diabetes Data & Trends. http://www.cdc.gov/diabetes/statistics/prev/national/figpersons.htm.

[2] Ceriello A. (2005). Postprandial hyperglycemia and diabetes complications. Diabetes.

[3] Monnier L., Colette C., Dunseath G.J., Owens D.R. (2007). The loss of postprandial glycemic control precedes stepwise deterioration of fasting with worsening diabetes. Diabetes Care.

[4] Cavalot F., Petrelli A., Traversa M., Bonomo K., Fiora E., Conti M., Anfossi G., Costa G., Trovati M. (2006). Postprandial blood glucose is a stronger predictor of cardiovascular events than fasting blood glucose in type 2 diabetes mellitus, particularly in women: Lessons from the San Luigi Gonzaga diabetes study. J. Clin. Endocrinol. Metab..

[5] Heo S.J., Hwang J.-H., Choi J.-I., Han J.S., Kim H.-J., Jeon Y.-J. (2009). Diphlorethohydroxycarmalol isolated from *Ishige okamurae*, a brown algae, a potent α-glucosidase and α-amylase inhibitor, alleviates postprandial hyperglycemia in diabetic mice. Eur. J. Pharmacol..

[6] Perfetti R., Barnett P.S., Mathur R., Egan J.E. (1998). Novel therapeutic strategies for the treatment of Type 2 diabetes. Diabetes/Metab. Res. Rev..

[7] Roy M.-C., Anguenot R., Fillion C., Beaulieu M., Bérubé J., Richard D. (2011). Effect of a commercially-available algal phlorotannins extract on digestive enzymes and carbohydrate absorption *in vivo*. Food Res. Int..

[8] Grabitske H.A., Slavin J.L. (2009). Gastrointestinal effects of low-digestible carbohydrates. Crit. Rev. Food Sci. Nutr..

[9] Etxeberria U., de la Garza A.L., Campión J., Martinez J.A., Milagro F.I. (2012). Antidiabetic effects of natural plant extracts via inhibition of carbohydrate hydrolysis enzymes with emphasis on pancreatic alpha amylase. Expert Opin. Ther. Targets.

[10] Van de Laar F.A. (2008). Alpha-glucosidase inhibitors in the early treatment of type 2 diabetes. Vasc. Health Risk Manag..

[11] Hanefeld M. (1998). The role of acarbose in the treatmet of non-insulin-dependent diabetes mellitus. J. Diabetes Complicat..

[12] Zhang J., Tiller C., Shen J., Wang C., Girouard G.S., Dennis D., Barrow C.J., Miao M., Ewart H.S. (2007). Antidiabetic properties of polysaccharide- and polyphenolic-enriched fractions from the brown seaweed *Ascophyllum nodosum*. Can. J. Physiol. Pharmacol..

[13] Paradis M.-E., Couture P., Lamarche B. (2011). A randomised crossover placebo-controlled trial investigating the effect of brown seaweed (*Ascophyllum nodosum* and *Fucus vesiculosus*) on postchallenge plasma glucose and insulin levels in men and women. Appl. Physiol. Nutr. Metab..

[14] Kim M.S., Kim J.Y., Choi W.H., Lee S.S. (2008). Effects of seaweed supplementation on blood glucose concentration, lipid profile, and antioxidant enzyme activities in patients with type 2 diabetes mellitus. Nutr. Res. Pract..

[15] Goñi I., Valdivieso L., Garcia-Alonso A. (2000). *Nori* seaweed consumption modifies glycemic response in healthy volunteers. Nutr. Res..

[16] Eom S.-H., Lee S.-H., Yoon N.Y., Jung W.-K., Jeon Y.-J., Kim S.-K., Lee M.-S., Kim Y.-M. (2012). α-Glucosidase- and α-amylase-inhibitory activities of phlorotannins from *Eisenia bicyclis*. J. Sci. Food Agric..

[17] Kim K.Y., Nam K.A., Kurihara H., Kim S.M. (2008). Potent α-glucosidase inhibitors purified from the red algae *Grateloupia elliptica*. Phytochemistry.

[18] Lee S.-H., Li Y., Karadeniz F., Kim M.-M., Kim S.-K. (2009). α-Glucosidase and α-amylase inhibitory activities of phloroglucinal derivatives from edible marine brown alga, *Ecklonia cava*. J. Sci. Food Agric..

[19] Lordan S., Smyth T.J., Soler-Vila A., Stanton C., Ross R.P. (2013). The α-amylase and α-glucosidase inhibitory effects of Irish seaweed extracts. Food Chem..

[20] Garza D. (2005). Common Edible Seaweeds in the Gulf of Alaska.

[21] Turner N.C., Bell M.A.M. (1973). The ethnobotany of the Southern Kwakiutl Indians of British Columbia. Econ. Bot..

[22] Turner N.J. (2003). The ethnobotany of edible seaweed (*Porphyra abbottae* and related species; Rhodophyta: Bangiales) and its use by First Nations on the Pacific Coast of Canada. Can. J. Bot..

[23] Wein E.E., Freeman M.M.R., Makus J.C. (1996). Use of and preference for traditional foods among the Belcher Island Inuit. Arctic.

[24] Nobmann E.D., Ponce R., Mattil C., Devereux R., Dyke B., Ebbesson S.O.E., Laston S., MacCluer J., Robbins D., Romenesko T. (2005). Dietary intakes vary with age among Eskimo adults of Northwest Alaska in the GOCADAN study, 2000–2003. J. Nutr..

[25] Bersamin A., Luick B.R., Ruppert E., STern J.S., Zidenberg-Cherr S. (2006). Diet quality among Yup’ik Eskimos living in rural communities is low: The Center for Alaska Native Health Research Pilot Study. J. Am. Diet. Assoc..

[26] Gahagan S., Silverstein J. (2003). Prevention and treatment of type 2 diabetes mellitus in children, with special emphasis on American Indian and Alaska Native children. Pediatrics.

[27] Ebbesson S.O.E., Risica P.M., Ebbesson L.O.E., Kennish J.M., Tejero E.M. (2005). Omega-3 fatty acids imrpove glucose tolerance and components of the metabolic syndrome in Alaskan Eskimos: The Alaska Siberia Project. Int. J. Circumpolar Health.

[28] Centers for Disease Control & Prevention (CDC) Racial and ethnic differences in diagnosed diabetes. http://www.cdc.gov/diabetes/pubs/estimates11.htm-4.

[29] Lindberg M.R., Lindstrom S.C. (2010). Field Guide to Seaweeds of Alaska.

[30] Kellogg J., Lila M.A. (2013). Chemical and *in vitro* assessment of Alaskan coastal vegetation antioxidant capacity. J. Agric. Food Chem..

[31] Steevensz A.J., MacKinnon S.L., Hankinson R., Craft C., Connan S., Stangel D.B., Melanson J.E. (2012). Profiling phlorotannins in brown macroalgae by liquid chromatography-high resolution mass spectrometry. Phytochem. Anal..

[32] Ferreres F., Lopes G., Gil-Izquierdo A., Andrade P.B., Sousa C., Mouga T., Valentão P. (2012). Phlorotannin extracts from Fucales characterized by HPLC-DAD-ESI-MS^n^: Approaches to hyaluronidase inhibitory capacity and antioxidant properties. Mar. Drugs.

[33] Glombitza K.W., Pauli K. (2003). Fucols and phlorethols from the brown alga *Scytothamnus australis* Hook. et Harv. (Chnoosporaceae). Bot. Mar..

[34] Grace M.H., Guzman I., Roopchand D.E., Moskal K., Cheng D.M., Pogrebnyak N., Raskin I., Howell A., Lila M.A. (2013). Stable binding of alternative protein-enriched food matrices with concentrated cranberry bioflavonoids for functional food applications. J. Agric. Food Chem..

[35] Xiao J., Kai G., Yamamoto K., Xiaoqing C. (2013). Advance in dietary polyphenols as α-glucosidases inhibitors: a review on structure-activity relationship aspect. Crit. Rev. Food Sci. Nutr..

[36] Spencer C.M., Cai Y., Martin R.E., Gaffney S.H., Goulding P.N., Magnolato D. (1988). Polyphenol complexation: Some thoughts and observations. Phytochemistry.

[37] Piparo E.L., Scheib H., Frei N., Williamson G., Grigorov M., Chou C.J. (2008). Flavonoids for controlling starch digestion: structural requirements for inhibiting human α-amylase. J. Med. Chem..

[38] Moon H.E., Islam M.N., Ahn B.R., Chowdhury S.S., Sohn H.S., Jung H.A., Choi J.S. (2011). Protein tyrosine phosphatase 1B and α-glucosidase inhibitory phlorotannins from edible brown algae, *Ecklonia stoloniera* and *Eisenia bicyclis*. Biosci. Biotechnol. Biochem..

[39] Koivikko R., Loponen J., Pihlaja K., Jormalainen V. (2007). High-performance liquid chromatographic analysis of phlorotannins from the brown alga *Fucus vesiculosus*. Phytochem. Anal..

[40] Kim T.K., Rioux L.E., Turgeon S.L. (2014). Alpha-amylase and alpha-glucosidase inhibition is diffeentially modulated by fucoidan obtained from *Fucus vesiculosus* and *Ascophyllum nodosum*. Phytochemistry.

